# Supplementary material for: Early-life medial pulvinar disruption drives schizophrenia-relevant prefrontal inhibitory and cognitive deficits in primates
Source: bioRxiv. 2026 Jul 24:2026.07.23.740162. Preprint. [Version 1] doi: 10.64898/2026.07.23.740162 (PMC13419517; doi:10.64898/2026.07.23.740162)
Supplement: 1 [file NIHPP2026.07.23.740162v1-supplement-1.pdf]

bioRxiv preprint doi: https://doi.org/10.64898/2026.07.23.740162; this version posted July 24, 2026. The copyright holder for this preprint (which was not certified by peer review) is the author/funder. This article is a US Government work. It is not subject to copyright under 17 USC 105 and is also made available for use under a CC0 license.

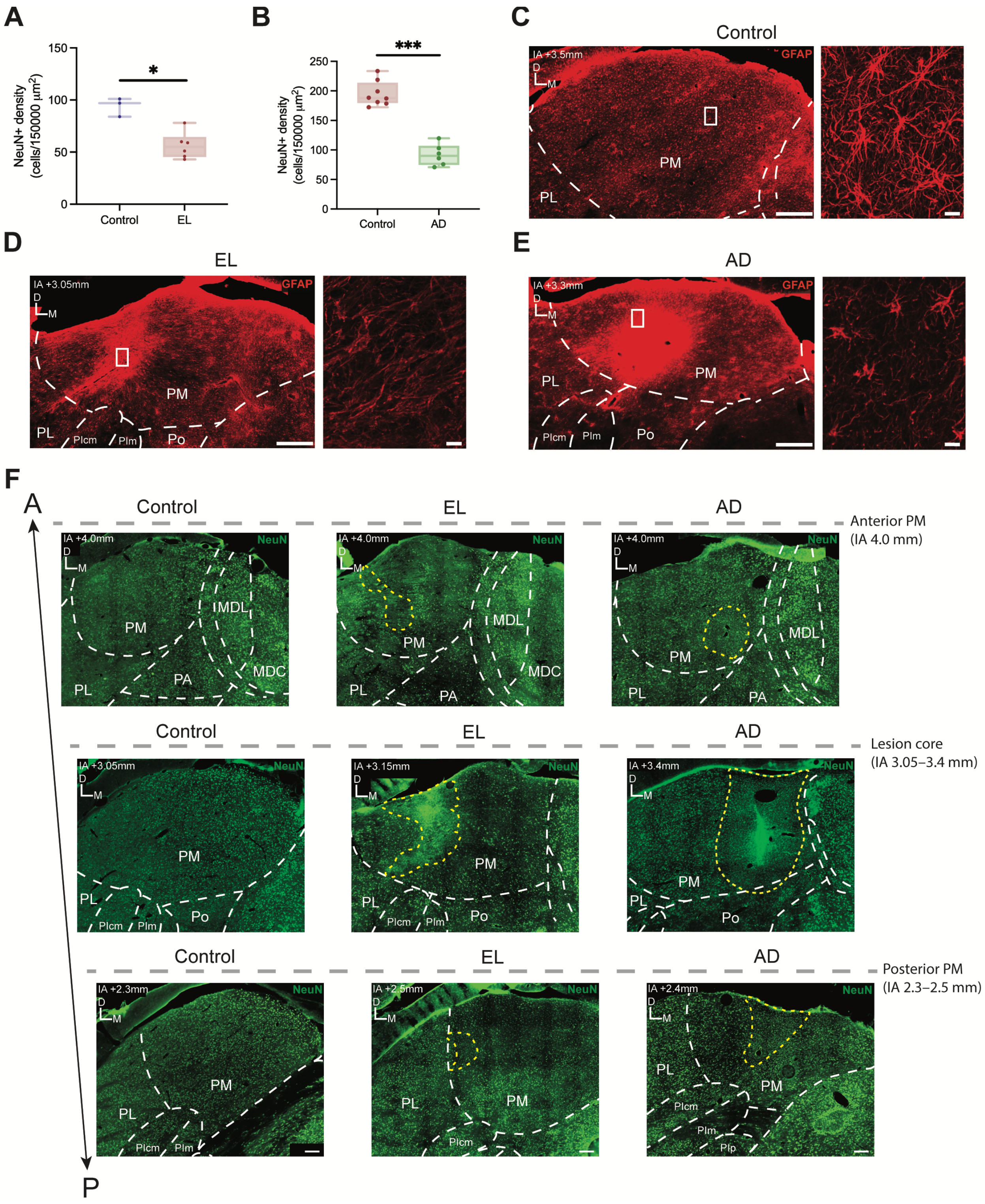

Supplementary Figure 1. Extended anatomical assessment of PM lesions sustained in early life or adulthood. Reductions in NeuN+ cell density after **A.** EL and **B.** AD. **C-E.** Qualitative assessment of reactive astrogliosis within lesion cores, depicting formation of a glial scar within the lesion core and reactive astrocytes in the penumbral area. Scale bar = 500 μm, 10 μm. **F.** Representative sections along the anteroposterior axis of animals from control, EL, and AD conditions showing the predominant restriction of lesions to PM. Scale bar = 200 μm. PM:

bioRxiv preprint doi: https://doi.org/10.64898/2026.07.23.740162; this version posted July 24, 2026. The copyright holder for this preprint (which was not certified by peer review) is the author/funder. This article is a US Government work. It is not subject to copyright under 17 USC 105 and is also made available for use under a CC0 license.

medial pulvinar; PL: lateral pulvinar; PIcm: inferior pulvinar (caudomedial division); PIm: inferior pulvinar (medial division); Po: posterior nuclei; MDL: mediodorsal nucleus (lateral division), MDC: mediodorsal nucleus (caudal division). * $p < 0.05$, *** $p < 0.001$.

bioRxiv preprint doi: https://doi.org/10.64898/2026.07.23.740162; this version posted July 24, 2026. The copyright holder for this preprint (which was not certified by peer review) is the author/funder. This article is a US Government work. It is not subject to copyright under 17 USC 105 and is also made available for use under a CC0 license.

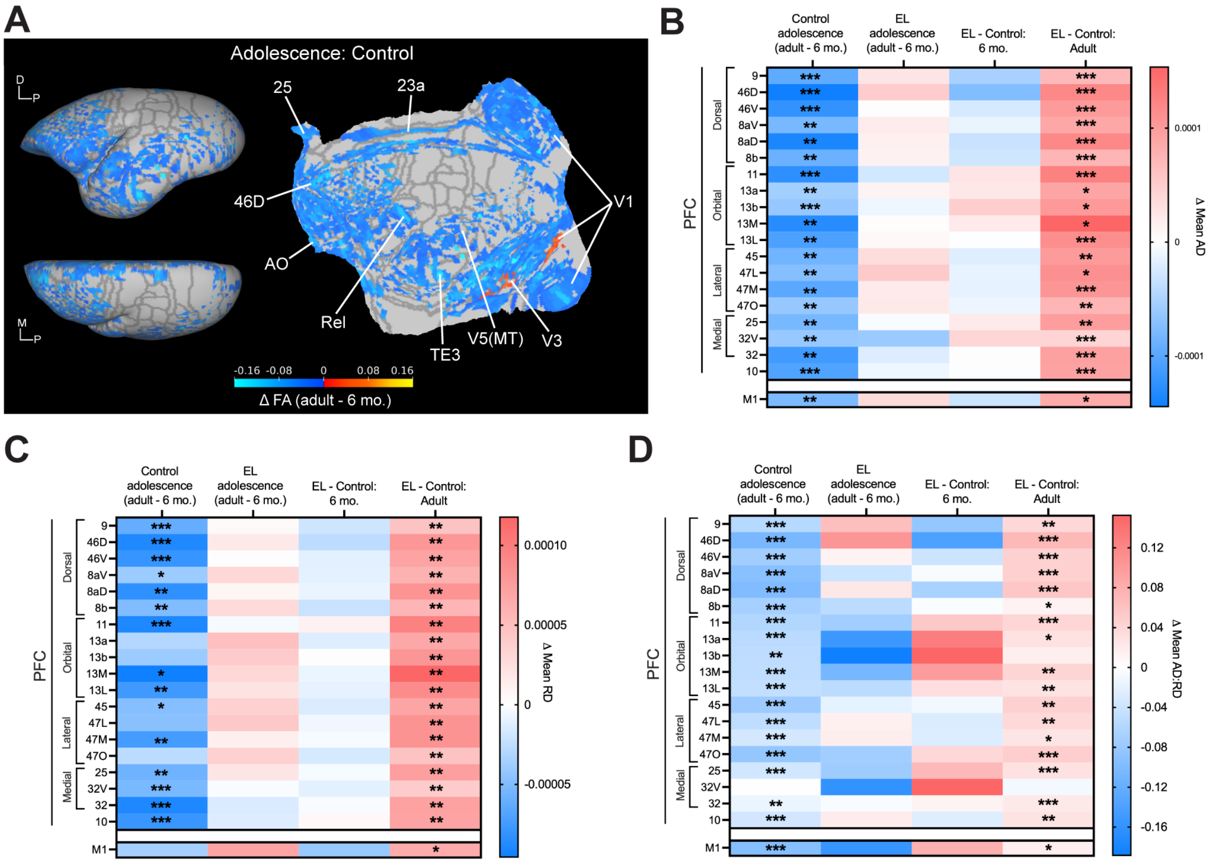

Supplementary Figure 2. Extended data revealing changes in cortical diffusivity over adolescence in EL compared to control animals. A. Statistical flatmap and volume renderings of voxel-wise FA differences in cortical gray matter between adulthood (>18 mo.) and 6 mo. in controls, indicative of typical adolescent maturation. Lower values indicate lesser FA in adulthood relative to 6 mo. **B-D.** ROI analysis of mean axial diffusivity (AD), radial diffusivity (RD), and AD:RD ratio across constituent areas of the PFC and M1 across age and condition. * $p < 0.05$, ** $p < 0.01$, *** $p < 0.001$.

bioRxiv preprint doi: https://doi.org/10.64898/2026.07.23.740162; this version posted July 24, 2026. The copyright holder for this preprint (which was not certified by peer review) is the author/funder. This article is a US Government work. It is not subject to copyright under 17 USC 105 and is also made available for use under a CC0 license.

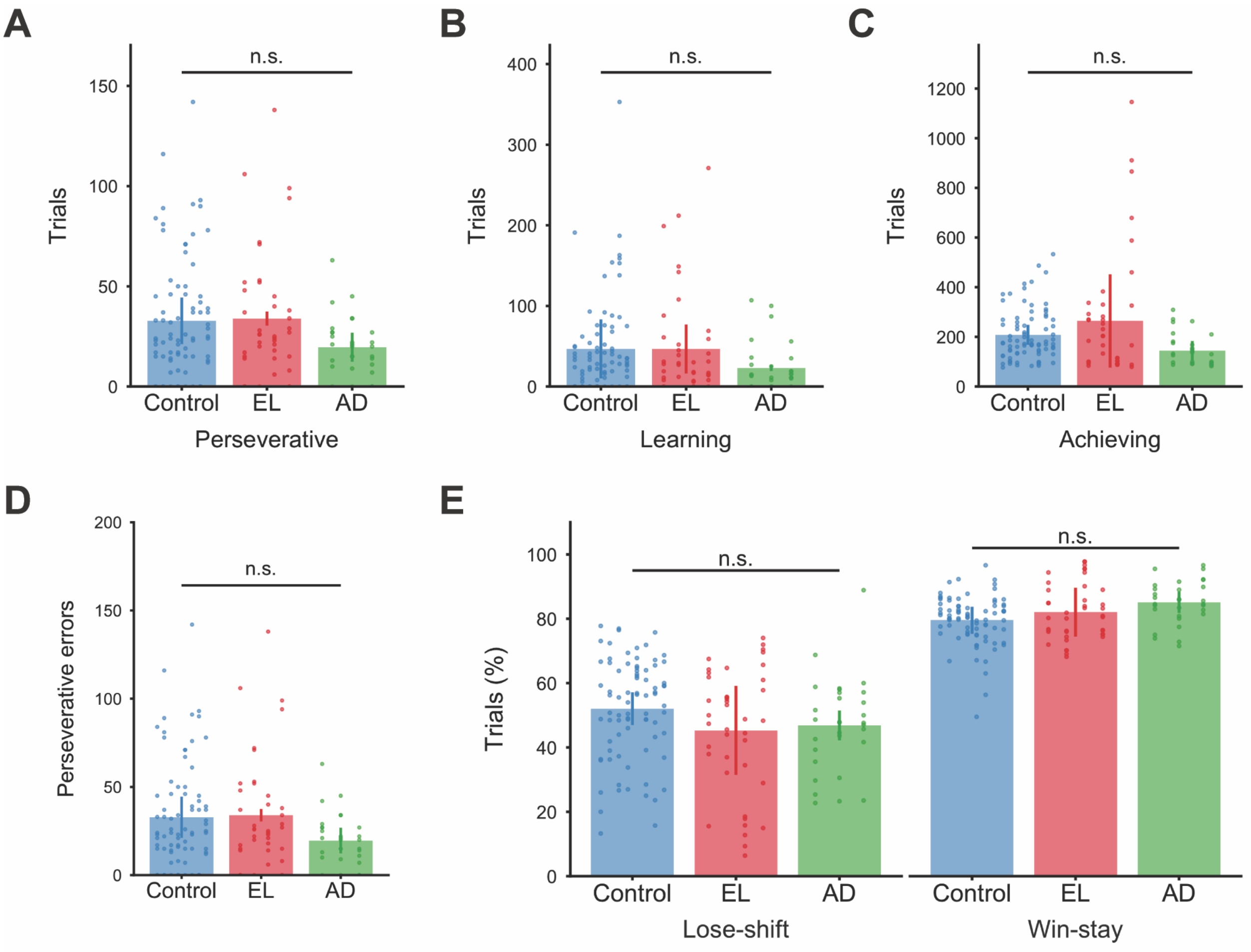

Supplementary Figure 3. Extended characterization of cognitive flexibility during reversal learning following early life or adult PM lesions. A-C. Number of trials performed across control, EL, and AD conditions in the perseverative, learning, and achieving stages of reversal learning, as defined by learning curve analysis. **D.** Number of errors committed during the perseverative stage across conditions. **E.** The proportions of lose-shift and win-stay response strategies performed in reversal learning sessions across conditions. Percentages are calculated based on the possible trial outcomes given the previous trial. Data points in the overlaid vertical scatter plot lines represent individual session performance for each animal.

bioRxiv preprint doi: https://doi.org/10.64898/2026.07.23.740162; this version posted July 24, 2026. The copyright holder for this preprint (which was not certified by peer review) is the author/funder. This article is a US Government work. It is not subject to copyright under 17 USC 105 and is also made available for use under a CC0 license.

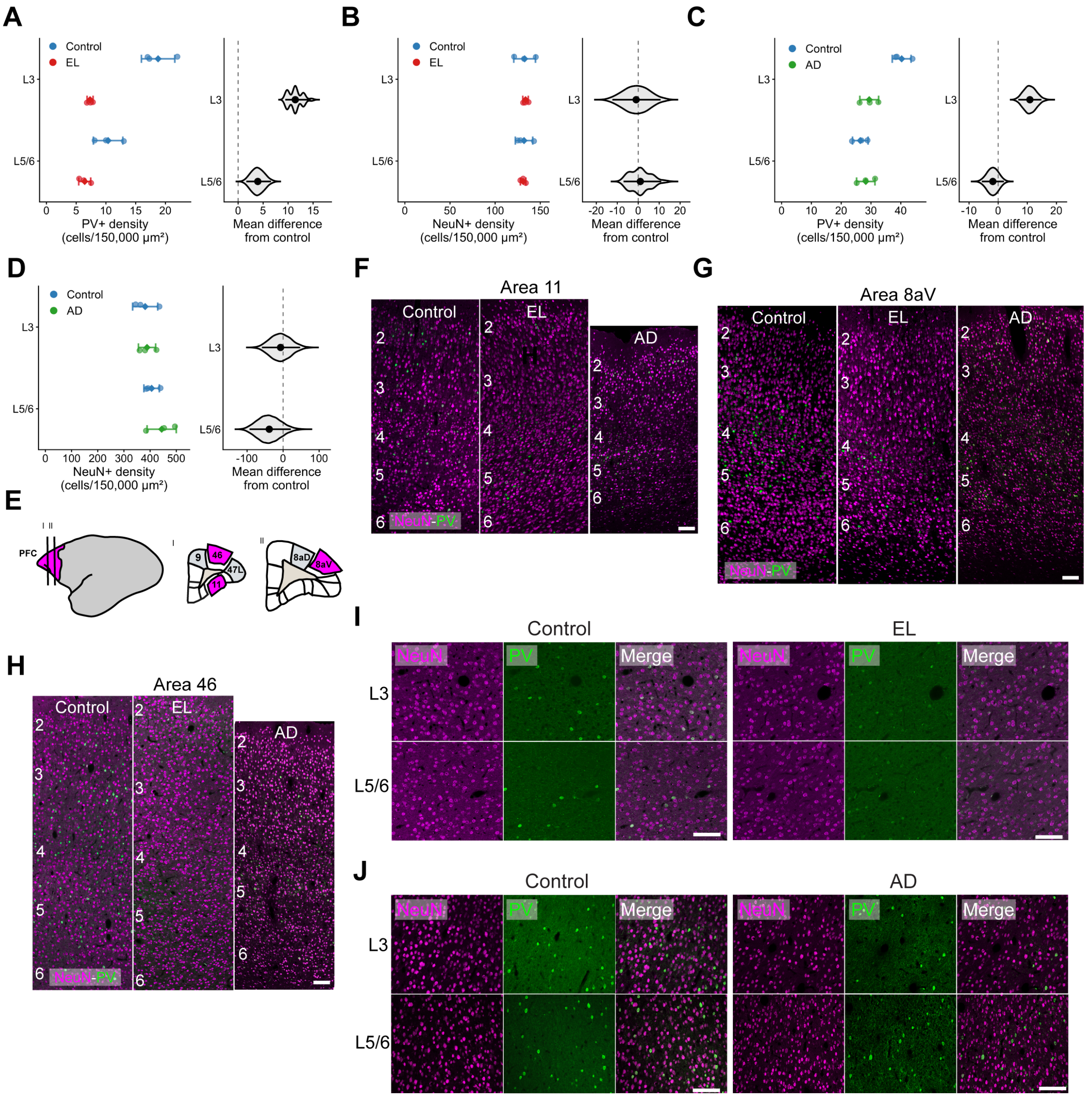

Supplementary Figure 4. Extended assessment of PV and NeuN expression in PFC following early life or adult PM lesions. A-D. Gardner-Altman estimation plots of raw cell density counts from both PV and NeuN immunolabelling in control, EL, and AD tissue. As the microscopy methods differed between EL and AD tissue, each cohort was compared with an independent cohort of method-matched control tissue. Left plots display mean +/- SD, right plots display mean difference +/- 95% bootstrapped CI and the bootstrapped distribution of the mean difference. **E.** Schematic of the PFC sections used for cell counting in the current figure. **F-H.** Laminar expression of NeuN/PV immunolabelling in control, EL, and AD tissue across 3 representative PFC areas. Scale bar = 100 µm. **I, J.** Representative images used for cell counting of PV/NeuN immunolabelling in layers 3 and 5/6. Scale bar = 100 µm.

bioRxiv preprint doi: https://doi.org/10.64898/2026.07.23.740162; this version posted July 24, 2026. The copyright holder for this preprint (which was not certified by peer review) is the author/funder. This article is a US Government work. It is not subject to copyright under 17 USC 105 and is also made available for use under a CC0 license.

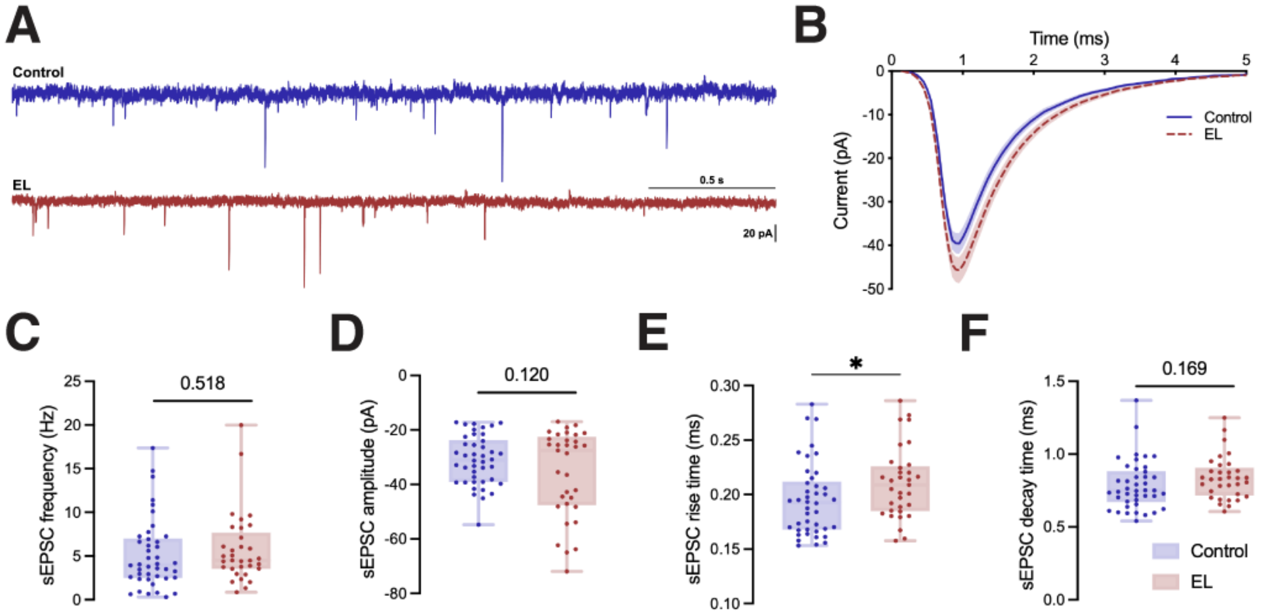

Supplementary Figure 5. Preservation of sEPSCs in PV-class interneurons of the PFC following early PM lesions. A. Representative sEPSC trace comparing control and EL conditions. **B.** Temporal dynamics of recorded sEPSCs. Data displays the mean of individual cell responses +/- SEM. **C-F.** Electrophysiological characteristics of recorded sEPSCs comparing control and EL conditions. * $p < 0.05$.

Supplementary Table 1. Experimental allocation of animals used in the study.

Animal	Intervention	Sex	Location	MRI: 6 mo.	MRI: Adult	Reversal Learning: Control/EL	Reversal Learning: AD	DMTS: Control/EL	DMTS: AD	ECoG: Control/EL	S5E2-targeted patch electrophysiology and anatomy	Immunohistochemistry: PFC PV/NeuN	Immunohistochemistry: Pulvinar NeuN	Immunohistochemistry: VGLUT2
TF77	Control	M	Facility 1	X										
TG53	Control	F	Facility 1	X										
TG54	Control	F	Facility 1	X										
TG57	Control	M	Facility 1	X										
TB51	Control	M	Facility 1			X								
TB52	Control	M	Facility 1			X					X		X	
TD45	Control	F	Facility 1			X								
TE12	Control	F	Facility 1								X			
TC75	Control	F	Facility 1									X	X	
TB20	Control	M	Facility 1									X	X	
TB54	Control	M	Facility 1									X	X	
173	Control	F	Facility 2									X	X	X
1234	Control	F	Facility 2									X	X	X
1804	Control	M	Facility 2									X	X	X
2133	Control	F	Facility 2			X		X		X				
2336	Control	M	Facility 2	X	X	X				X				
M181	Control	F	Facility 3		X									
M182	Control	F	Facility 3		X									
M197	Control	F	Facility 3		X									
M198	Control	F	Facility 3		X									
M199	Control	F	Facility 3		X									
M200	Control	M	Facility 3		X									
M177	Control	M	Facility 3		X									
S12	Control/AD	M	Facility 1			X	X	X	X	X				
TE11	Control/AD	F	Facility 1			X	X	X	X	X				
2334	Control/AD	F	Facility 1 & 2	X	X	X	X	X	X			X	X	
TE27	AD	M	Facility 1									X	X	
TB30	AD	M	Facility 1									X	X	
TF75	EL	M	Facility 1	X										
TE47	EL	F	Facility 1							X				
2389	EL	F	Facility 1 & 2								X			
2391	EL	M	Facility 1 & 2								X			
2310	EL	M	Facility 2	X	X									
2280	EL	M	Facility 2	X	X	X		X						
2237	EL	M	Facility 2		X	X								
2223	EL	M	Facility 2		X	X		X		X				
2279	EL	M	Facility 2		X	X		X		X				
2193	EL	F	Facility 2									X	X	X
2083	EL	M	Facility 2									X	X	X
2222	EL	F	Facility 2									X	X	X

Supplementary Table 2. Electrophysiological parameters of S5E2+ cells.

Parameter	Control	EL	Permutation test p-value
RMP (mV)	-62.0 ± 1.1 (n = 51)	-59.5 ± 1.0 (n = 47)	0.099
Spont. AP Frequency (Hz)	1.46 ± 0.64 (n = 44)	5.45 ± 1.94 (n = 37)	0.038 (*)
Tau (ms)	10.6 ± 0.5 (n = 52)	13.6 ± 1.0 (n = 47)	0.001 (**)
R_{in} (MΩ)	124.6 ± 6.5 (n = 50)	187.2 ± 13.6 (n = 45)	0.0005 (****)
C_{in} (pF)	95.2 ± 6.7 (n = 50)	80.2 ± 5.7 (n = 45)	0.087
Sag (mV)	7.40 ± 0.45 (n = 44)	7.08 ± 0.48 (n = 48)	0.633
Sag Index	0.77 ± 0.01 (n = 51)	0.78 ± 0.01 (n = 48)	0.568
Max. Frequency (Hz)	249 ± 9 (n = 51)	216 ± 11 (n = 47)	0.022 (*)
Inst. Frequency (Hz)	313 ± 10 (n = 51)	286 ± 13 (n = 47)	0.113
Accommodation	0.76 ± 0.01 (n = 51)	0.72 ± 0.02 (n = 47)	0.043 (*)
AP Threshold (mV)	-41.5 ± 0.7 (n = 51)	-40.7 ± 0.7 (n = 47)	0.452
AP Amplitude (mV)	47.8 ± 1.1 (n = 51)	49.4 ± 1.4 (n = 47)	0.360
AP Width (ms)	0.36 ± 0.01 (n = 51)	0.43 ± 0.02 (n = 47)	0.002 (**)
Max Upstroke (mV/ms)	253.6 ± 8.8 (n = 51)	234.8 ± 8.6 (n = 47)	0.128
Max Downstroke (mV/ms)	-177.4 ± 7.8 (n = 51)	-154.5 ± 8.2 (n = 47)	0.045 (*)
AHP Amplitude (mV)	-19.9 ± 0.7 (n = 48)	-20.1 ± 0.6 (n = 47)	0.883
AHP Time (ms)	1.93 ± 0.14 (n = 48)	3.29 ± 0.98 (n = 47)	0.039 (*)
sEPSC Frequency (Hz)	5.17 ± 0.62 (n = 42)	5.78 ± 0.70 (n = 33)	0.519
sEPSC Amplitude (pA)	-31.1 ± 1.4 (n = 42)	-35.7 ± 2.8 (n = 33)	0.120
sEPSC Rise Time (ms)	0.195 ± 0.005 (n = 42)	0.211 ± 0.006 (n = 33)	0.047 (*)
sEPSC Decay Time (ms)	0.785 ± 0.026 (n = 42)	0.837 ± 0.026 (n = 33)	0.169

bioRxiv preprint doi: https://doi.org/10.64898/2026.07.23.740162; this version posted July 24, 2026. The copyright holder for this preprint (which was not certified by peer review) is the author/funder. This article is a US Government work. It is not subject to copyright under 17 USC 105 and is also made available for use under a CC0 license.

Supplementary Table 3. Primary and secondary antibodies used for immunohistochemistry.

Antigen	Figure abbreviation	Host species	Dilution	Source	Catalog ID	RRID
**Primary**						
Neuronal nuclear antigen	NeuN	Rabbit	1:1000	Millipore-Sigma	ABN78	AB_10807945
Parvalbumin	PV	Rabbit	1:1000	Swant	PV28	AB_2315235
Parvalbumin	PV	Mouse	1:1000	Swant	PV235	AB_3698492
Parvalbumin	PV	Mouse	1:1000	Millipore-Sigma	P3088	AB_477329
Glial fibrillary acidic protein	GFAP	Mouse	1:1000	Millipore-Sigma	MAB360	AB_11212597
Vesicular glutamate transporter 2	VGLUT2	Mouse	1:500	Millipore-Sigma	MAB5504	AB_2187552
Calbindin	Calb	Mouse	1:1000	Swant	CB300	AB_10000347
DsRed	S5E2	Rabbit	1:1000	Takara	632496	AB_10013483
**Secondary**						
Donkey anti-mouse Alexa Fluor 488	-	Donkey	1:800	Thermo Fisher	A21202	AB_141607
Donkey anti-mouse Alexa Fluor 647	-	Donkey	1:800	Thermo Fisher	A31571	AB_162542
Donkey anti-rabbit Alexa Fluor 488	-	Donkey	1:800	Thermo Fisher	A21206	AB_2535792
Donkey anti-rabbit Alexa Fluor 594	-	Donkey	1:800	Thermo Fisher	A21207	AB_141637
Goat anti-rabbit Alexa Fluor 488	-	Goat	1:800	Thermo Fisher	A11008	AB_143165
Goat anti-rabbit Alexa Fluor 488	-	Goat	1:1000	Thermo Fisher	A11034	AB_2576217
Goat anti-mouse Alexa Fluor 647	-	Goat	1:800	Thermo Fisher	A21235	AB_2535804
Goat anti-rabbit Alexa Fluor 555	-	Goat	1:1000	Thermo Fisher	A21429	AB_2535850
**Other conjugate**						

bioRxiv preprint doi: https://doi.org/10.64898/2026.07.23.740162; this version posted July 24, 2026. The copyright holder for this preprint (which was not certified by peer review) is the author/funder. This article is a US Government work. It is not subject to copyright under 17 USC 105 and is also made available for use under a CC0 license.

Streptavidin Alexa Fluor 555	-	-	1:500	Thermo Fisher	S32355	AB_2571525
